# Association between LDL-C/HDL-C ratio and long-term carotid plaque risk in middle-aged and elderly rural populations: a prospective population study

**DOI:** 10.3389/fmed.2026.1771449

**Published:** 2026-02-17

**Authors:** Yingzhe Shao, Juan Hao, Hailu Zhang, Yixin Chen, Jun Tu, Xianjia Ning, Yan Li

**Affiliations:** 1 Department of Neurology, Tianjin Medical University General Hospital, Tianjin, China; 2 Department of Oncology, Tianjin Jizhou People’s Hospital, Tianjin, China; 3 School of Basic Medical Sciences, Tianjin Medical University, Tianjin, China; 4 Institute of Clinical Epidemiology & Evidence-Based Medicine, Tianjin Jizhou People’s Hospital, Tianjin, China; 5 Laboratory of Epidemiology, Tianjin Neurological Institute, Tianjin, China; 6 Key Laboratory of Post-Neuroinjury Neuro-repair and Regeneration in Central Nervous System, Ministry of Education and Tianjin City, Tianjin Neurological Institute, Tianjin, China

**Keywords:** atherosclerosis, cardiovascular risk, carotid plaque, LDL-C/HDL-C ratio, rural population

## Abstract

**Background:**

Atherosclerotic cardiovascular and cerebrovascular diseases are still the main cause of global incidence rate and mortality. The LDL-C/HDL-C ratio (LHR) has been identified as a potential biomarker for cardiovascular risk. However, the relationship between it and the long-term risk of carotid plaques is not yet clear, especially in low-income populations in China.

**Methods:**

This prospective cohort study included adults aged ≥45 years without carotid plaque at baseline from low-income rural areas of Tianjin, China. Baseline characteristics were collected in 2014, and follow-up data were obtained in 2019. The primary outcome was the development of new carotid plaques, assessed using carotid ultrasound. The relationship between the LDL-C/HDL-C ratio (LHR) and new carotid plaques was analyzed using multifactorial logistic regression, with the presence or absence of new-onset plaques as the dependent variable. Additionally, we utilized restricted cubic spline (RCS) regression to visually present the potential nonlinear relationship between LHR and the risk of carotid plaque.

**Results:**

Over the six-year follow-up period, 606 participants (38.3%) developed new carotid plaques. Higher LHR was significantly associated with an increased risk of new carotid plaques, with each unit increase in LHR corresponding to a 35.9% higher risk (OR = 1.359, 95% CI: 1.180–1.566, *p* < 0.001). The RCS curve indicated a non-linear positive association between LHR and the likelihood of carotid plaques (*p* for non-linearity = 0.019), with an optimal cut-off at 1.257. Logistic regression analysis confirmed that LHR > 1.257 was linked to increased odds of carotid plaques in both unadjusted (OR: 1.80, *p* < 0.001) and adjusted models (OR: 1.835, *p* < 0.001), with LHR ≤ 1.257 serving as the reference. In subgroup analysis, all subgroups consistently demonstrated a significant association between increased LHR (all OR > 1).

**Conclusion:**

The research results indicate that there is a non-linear positive correlation between LHR and the long-term risk of carotid plaques in middle-aged and elderly populations, suggesting that LHR may be an effective indicator for screening carotid plaques in grassroots middle-aged and elderly populations.

## 1 Introduction

Atherosclerotic cardiovascular and cerebrovascular diseases constitute a major global health burden. They are responsible for approximately 18.6 million deaths and 34.4 million disability-adjusted life years (DALYs) annually, making them among the leading causes of mortality and long-term disability worldwide. Despite advancements in medical care and preventive strategies, the incidence and overall burden of these conditions continue to rise (1, 2).

Among them, lower income is associated with higher CVD morbidity and mortality (3, 4). A recent study showed that the prevalence of carotid plaque was as high as 21.0% in China (5). Age is an important risk factor for carotid plaque occurrence. As a risk factor for cardiovascular events, carotid plaque is significantly associated with cardiovascular disease events, myocardial infarction and all-cause mortality (6). Therefore, early prediction of carotid plaque can help to reduce the risk of cardiovascular events and reduce the burden on society.

High LDL-C and low HDL-C are thought to be highly associated with atherosclerosis (7). Studies have shown that the LDL-C/HDL-C ratio (LHR) has better results in predicting cardiovascular disease compared to LDL-C or HDL-C alone (8, 9). An increase in the LHR is associated with a blockage of HDL maturation process and thus with the development of atherosclerosis (7). Several studies have shown that the LHR can predict the development of carotid plaque. A study on type 2 diabetic patients showed that LHR was positively associated with CIMT and the presence of carotid plaques in men (10). A cross-sectional study of obese adults in Xinjiang Uyghur showed that the LHR was associated with carotid plaque (11). A study on patients with coronary artery disease showed that high LHR was associated with the risk of carotid plaque in patients with coronary artery disease (12). Another cohort study on Nanjing, China, with a follow-up of 1.05 years, showed that a high LHR accelerated the development of carotid plaques, especially in older men (13). However, although it has been demonstrated that the LHR index is associated with the development of carotid plaques, most of the studies have been conducted based on hospital cases and are mostly cross-sectional, and there is a lack of long-term prospective cohort studies on the relevant populations, in particular, the relationship between the LHR and the prevalence of carotid plaques in low-income people in rural areas is not yet clear.

Therefore, the present study is a 6-year prospective cohort study based on low-income rural residents in Tianjin, aiming to investigate the association between LHR and new-onset carotid plaques in rural low-income residents, and to explore its predictive role in different characteristics of the population through stratified analysis.

## 2 Methods

### 2.1 Study population

This is a prospective study based on low-income community residents, and the atherosclerosis cohort consisted of low-income individuals from the Tianjin Brain Study in China (14, 15); permanent residents aged ≥45 years were included in this study. The atherosclerosis cohort initially recruited 3,992 participants in 2014, with inclusion criteria of (1) Greater than or equal to 45 years of age. (2) Absence of carotid plaque on carotid ultrasound in 2014. (3) Having both 2014 and 2019 carotid ultrasound results and baseline LDL-C and HDL-C data. (4) Those who do not have major illnesses such as liver disease, kidney disease, or mental illness.

The study complied with the Declaration of Helsinki and was approved by the Ethics Committee of Tianjin Medical University General Hospital. All participants provided written informed consent.

### 2.2 Data collection

A pre-designed questionnaire, which was described previously, was used to collect patient information. Of these, socio-demographic and clinical data collected included name, gender, age, education, lifestyle, history of diabetes, history of hypertension. Trained epidemiological researchers conducted face-to-face interviews using pre-designed standardized questionnaires to collect data. Participants were grouped by age: 45–54 years group and 55–64 years and ≥65 years groups. In addition, lifestyle information included smoking status (never smoked, ever smoked, or currently smoked) and alcohol consumption status (never drank, ever drank, or currently drank). Laboratory parameters included systolic blood pressure, diastolic blood pressure, fasting blood glucose, serum total cholesterol (TC), serum total triglycerides (TG), high-density lipoprotein cholesterol (HDL-C), and low-density lipoprotein cholesterol (LDL-C). All blood samples used for biochemical analysis were strictly collected under fasting conditions.

Hypertension was defined as a previous history of hypertension and three blood pressure averages in the current study of systolic blood pressure greater than or equal to 140 mm Hg or diastolic blood pressure greater than or equal to 90 mm Hg. Diabetes mellitus was defined as a previous history of diabetes mellitus or a fasting glucose level of ≥7.0 mmol/L in the current study. Cigarette smoking was defined as having smoked at least one cigarette per day for more than 1 year. Smoking cessation was defined as quitting smoking for more than 1 year or smoking less than 1 cigarette per month in the 1 year prior to the questionnaire. Alcohol consumption was defined as drinking alcohol at least once a week and having a total alcohol intake of more than 50 mL per week for more than 6 months. Alcohol abstinence was defined as abstinence from alcohol for more than 1 year or intake of less than 12 grams of alcohol per month in the 1 year prior to the questionnaire. Individuals categorized as drinkers had consumed alcohol more than once a week for more than 1 year with ≥12 g of alcohol intake per occasion (16).

### 2.3 Physical and biochemical examinations

Parameters measured included height (meters), weight (kilograms), and blood pressure (millimeters of mercury). Specific measurements are detailed in a previous publication (16). Blood samples were collected from participants in a fasting state. Fasting blood glucose and serum concentrations of TC, TG, HDL-C and LDL-C were determined. In addition, carotid ultrasound was performed by a trained physician. Body mass index (BMI) was calculated as weight in kilograms divided by the square of height in square meters. All measurements were recorded by the same follow-up person to minimize systematic errors. Based on the body mass index (BMI) values, participants were categorized into four groups: low weight group, BMI < 18.5 kg/m^2^, normal weight group, 18.5 kg/m^2^ ≤ BMI < 24.00 kg/m^2^, overweight group, 24 kg/m^2^ ≤ BMI < 28.00 kg/m^2^, and obese group, BMI ≥ 28.00 kg/m^2^. TyG index, which is a composite index composed of triglyceride and glucose, calculated using the following formula: In [serum triglyceride (mg/dl) × serum blood glucose (mg/dl)/2] (17).

### 2.4 Ultrasonic measurement

IMT was measured in real time using an ultrasound instrument equipped with electronic calipers. Trained sonographers performed carotid ultrasound without knowledge of basic participant data. The proximal and distal walls and bifurcations of the common carotid, internal carotid, and external carotid arteries were scanned in anterior, lateral, and posterior projections using a standardized protocol. Carotid plaque was defined as follows: (1) localized carotid intima-media thickness ≥1.5 mm or (2) nearby protrusion extending into the lumen and exceeding the surrounding intima-media thickness by >50% (18). The ultrasound examination personnel were unaware of the biochemical test results and other information of the research subjects.

### 2.5 Statistical analysis

Continuous variables were expressed as mean ± standard deviation, and comparisons between groups were made using *t*-tests. Categorical variables were expressed as percentages, and the chi-square test was used for between-group comparisons. The relationship between the LHR and new carotid plaques was analyzed using multifactorial logistic regression, with the presence or absence of new-onset plaques as the dependent variable. Variables with a *p*-value <0.05 in univariate analysis were included in the multifactorial model. All patients were further divided into four categories according to their IQR for LHR mean values, including the Q1 (LHR ≤ 1.356), Q2 (1.356 < LHR ≤ 1.842), Q3 (1.842 < LHR ≤ 2.393) and Q4 (LHR > 2.393) categories, to further explore the relationship between different LHR categories and outcome indicators. Additionally, we utilized restricted cubic spline (RCS) regression to visually present the potential nonlinear relationship between LHR and the risk of carotid plaque. The number of nodes in the RCS model was determined to be 3 based on the minimum Akaike Information Criterion (AIC) comparison. The node positions were set at the 10th, 50th (median), and 90th percentiles of the distribution of this variable, and the reference point was set at the median. The model results were interpreted as the effect changes relative to this reference point. This setting effectively avoids overfitting in data-sparse areas while providing sufficient flexibility for fitting. Analyses were stratified by sex, age, hypertension, and diabetes status, with results expressed as adjusted odds ratio (OR) and 95% confidence intervals (95% CI). Statistical analyses were performed using SPSS version 27.0, and two-tailed *p*-values <0.05 were considered significant. Restricted cubic spline plots were drawn using R4.4.0 software.

## 3 Results

**Figure 1:**
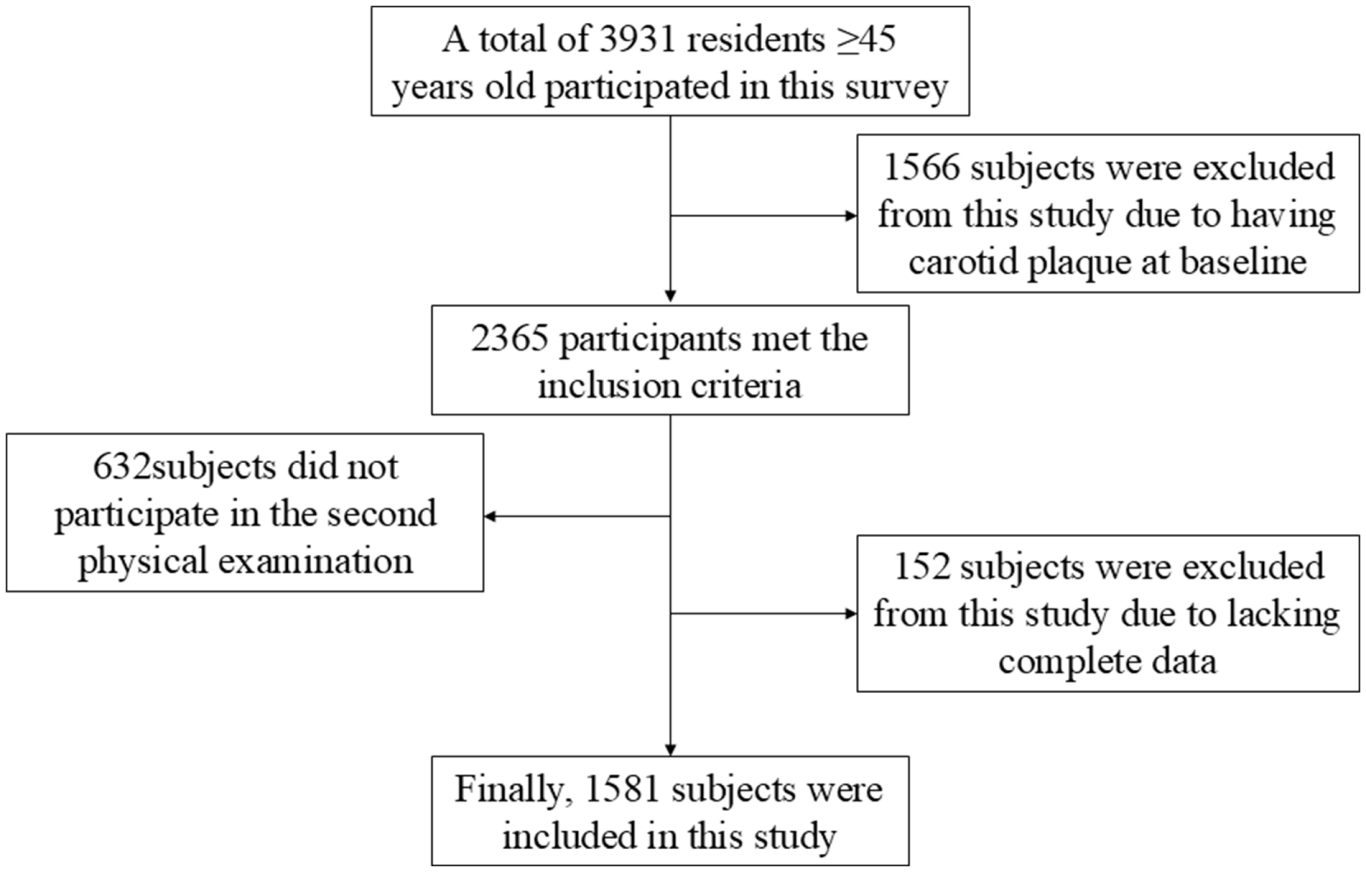
Flow chart of participants’ selection. This figure showed that 3,931 individuals from low-income rural areas of Tianjin were initially screened in 2014. There were 2,365 individuals, who meet the selection criteria of this study, after exclusion of individuals with existing carotid plaques detected by ultrasound in 2014 (*n* = 1,566). Finally, 1,581 participants were included in the final analysis after excluding those with incomplete data or lost to follow-up (*n* = 784).

In 2014, a preliminary screening was conducted on 3,931 low-income rural residents age ≥45 years in Tianjin. After excluding those who had detected carotid plaque through ultrasound examination in 2014 (*n* = 1,566) and those who did not participate in the second examination (*n* = 632), 1733 cases met the selection criteria for this study. Finally, 1581 participants were included in the final analysis after excluding those with incomplete data (*n* = 152; Figure 1).

### 3.1 Demographic characteristics

A total of 1,581 investigators were enrolled in this study, 35.2% were male and the mean age was 58.42 years, out of which 606 (38.33%) had new carotid plaques, 43.9% of them were male and the mean age was 60.58 years (Table 1).

**Table 1:** Baseline characteristics of the study cohort.

Characteristics	Men	Women	Total
Case, *n* (%)	557 (35.2)	1,024 (64.8)	1,581 (100)
Age^a^, years	59.55 ± 8.07	57.81 ± 7.76	58.42 ± 7.91
Age groups, *n* (%)			
45–54	152 (27.3)	367 (35.8)	519 (32.8)
55–64	255 (45.8)	454 (44.3)	709 (44.8)
≥65	150 (26.9)	203 (19.8)	353 (22.3)
Education level^a^, year	6.60 ± 2.87	4.46 ± 3.66	5.21 ± 3.55
BMI^a^, kg/m^2^	25.25 ± 3.28	26.12 ± 3.66	25.81 ± 3.55
BMI groups^a^, *n* (%)			
Low weight	4 (0.7)	8 (0.8)	12 (0.8)
Normal weight	201 (36.2)	278 (27.2)	479 (30.4)
Overweight	242 (43.5)	472 (46.2)	714 (45.2)
Obesity	109 (19.6)	264 (25.8)	373 (23.6)
Smoke status, *n* (%)			
Never smoking	106 (19)	957 (94.1)	1,063 (67.5)
Current smoking	244 (43.8)	29 (2.9)	273 (17.3)
Ever smoking	207 (37.2)	31 (3.0)	238 (15.1)
Alcohol consumption, *n* (%)			
Never drinking	142 (25.5)	979 (95.8)	1,121 (71)
Current drinking	304 (54.6)	23 (2.3)	327 (20.7)
Ever drinking	111 (19.9)	20 (2.0)	131 (8.3)
Hypertension, *n* (%)			
No	182 (32.7)	297 (29.0)	479 (30.3)
Yes	375 (67.3)	727 (71.0)	1,102 (69.7)
Diabetes, *n* (%)			
No	457 (82)	832 (81.3)	1,289 (81.5)
Yes	100 (18)	192 (18.8)	292 (18.5)
SBP^a^, mmHg	145.59 ± 19.30	143.31 ± 20.84	144.11 ± 20.33
DBP^a^, mmHg	89.14 ± 10.88	85.55 ± 11.19	86.81 ± 11.21
FPG^a^, mmol/L	5.88 ± 1.38	5.81 ± 1.31	5.83 ± 1.33
TC^a^, mmol/L	4.49 ± 0.91	4.95 ± 0.99	4.79 ± 0.99
TG^a^, mmol/L	1.54 ± 1.54	1.72 ± 1.00	1.66 ± 1.22
HDL-C^a^, mmol/L	1.40 ± 0.47	1.48 ± 0.41	1.45 ± 0.43
LDL-C^a^, mmol/L	2.43 ± 0.78	2.69 ± 0.87	2.60 ± 0.85
Newly developed plaques, *n* (%)	266 (47.8)	340 (33.2)	606 (38.3)
LHR^a^	1.89 ± 0.81	1.93 ± 0.75	1.92 ± 0.77
TyG	8.67 ± 0.59	8.82 ± 0.61	8.77 ± 0.60

a Continuous variables were expressed as mean ± standard deviation. BMI, body mass index; SBP, systolic blood pressure; DBP, diastolic blood pressure; FPG, fasting plasma glucose; TC, total cholesterol; TG, triglycerides; LDL-C, low-density lipoprotein cholesterol; HDL-C, high-density lipoprotein cholesterol; LHR, LDL-C/HDL-C ratio. TyG, triglyceride-glucose index.

### 3.2 The associated factors of the new carotid plaque incidence in the univariate analysis

Univariate analysis revealed significant associations between several factors and the incidence of new carotid plaques. Participants with new carotid plaques were older (60.58 ± 8.05 years) compared to those without plaques (57.08 ± 7.52 years) (*p* < 0.001). A higher proportion of men, current smokers, ever smokers, current drinkers, and ever drinkers developed new carotid plaques compared to their counterparts (*p* < 0.001). Additionally, participants with new plaques had higher SBP, DBP, FPG, LDL-C, and LHR levels, while HDL-C levels were lower compared to those without new plaques (Table 2).

**Table 2:** Univariate analysis of the relationship between carotid artery plaque and influencing factors.

Characteristics	No newly developed plaques	Newly developed plaques	*p* Value
Case, *n* (%)	975 (61.7)	606 (38.3)	
Men, *n* (%)	291 (29.8)	266 (43.9)	<0.001
Age^a^, years	57.08 ± 7.52	60.58 ± 8.05	<0.001
Age groups, *n* (%)			<0.001
45–54	380 (39)	139 (22.9)	
55–64	424 (43.5)	285 (47)	
≥65	171 (17.5)	182 (30.0)	
BMI^a^, kg/m^2^	25.77 ± 3.59	25.87 ± 3.50	0.586
BMI groups^a^, *n* (%)			0.924
Low weight	8 (0.8)	4 (0.7)	
Normal weight	297 (30.5)	182 (30.1)	
Overweight	443 (45.5)	271 (44.8)	
Obesity	225 (23.1)	148 (24.5)	
Smoke status, *n* (%)			<0.001
Never smoking	701 (72.3)	362 (59.8)	
Current smoking	151 (15.6)	122 (20.2)	
Ever smoking	117 (12.1)	121 (20.0)	
Alcohol consumption, *n* (%)			<0.001
Never drinking	729 (74.9)	392 (64.7)	
Current drinking	183 (18.8)	144 (23.8)	
Ever drinking	61 (6.3)	70 (11.6)	
Hypertension, *n* (%)			<0.001
No	350 (35.9)	129 (21.3)	
Yes	625 (64.1)	477 (78.7)	
Diabetes, *n* (%)			<0.001
No	831 (85.2)	458 (75.6)	
Yes	144 (14.8)	148 (24.4)	
SBP^a^, mmHg	141.62 ± 20.20	148.12 ± 19.92	<0.001
DBP^a^, mmHg	86.22 ± 11.36	87.78 ± 10.89	0.007
FPG^a^, mmol/L	5.72 ± 1.25	6.02 ± 1.44	<0.001
TC^a^, mmol/L	4.78 ± 1.00	4.81 ± 0.97	0.518
TG^a^, mmol/L	1.64 ± 1.24	1.68 ± 1.19	0.484
HDL-C^a^, mmol/L	1.49 ± 0.45	1.39 ± 0.38	<0.001
LDL-C^a^, mmol/L	2.56 ± 0.86	2.66 ± 0.83	0.024
LHR^a^	1.84 ± 0.76	2.03 ± 0.77	<0.001
TyG	8.70 ± 0.59	8.81 ± 0.61	0.034

a Continuous variables were expressed as mean ± standard deviation. BMI, body mass index; SBP, systolic blood pressure; DBP, diastolic blood pressure; FPG, fasting plasma glucose; TC, total cholesterol; TG, triglycerides; LDL-C, low-density lipoprotein cholesterol; HDL-C, high-density lipoprotein cholesterol; LHR, LDL-C/HDL-C ratio.

### 3.3 The associated factors of the new carotid plaque incidence in the multivariate analysis

Table 3 shows the results of multivariate analysis of factors influencing carotid artery plaque. LHR was significantly associated with carotid plaque (OR = 1.359, 95% CI: 1.180–1.566, *p* < 0.001). Women had a lower risk of plaque compared to men (OR = 0.676, 95% CI: 0.461–0.991, *p* = 0.045). Smoking status and alcohol consumption were not significantly related to carotid plaque (*p* > 0.05). Age groups showed a significant association: those aged 55–64 (OR = 2.375, 95% CI: 1.751–3.221, *p* < 0.001) and ≥65 (OR = 1.618, 95% CI: 1.254–2.089, *p* < 0.001) had a higher risk compared to those aged 45–54. Higher fasting plasma glucose (FPG) and systolic blood pressure (SBP) were also associated with increased risk (FPG: OR = 1.009, *p* = 0.001; SBP: OR = 1.095, *p* = 0.044). However, the TyG index was not significantly related to carotid plaque (*p* = 0.701).

**Table 3:** Multivariate analysis of the relationship between carotid artery plaque and influencing factors.

Characteristics	Reference	OR (95%CI)	*p* value
LHR		1.359 (1.180, 1.566)	<0.001
Gender	Men		
Women		0.676 (0.461, 0.991)	0.045
Smoke status	Never smoking		
Current smoking		1.298 (0.866, 1.946)	0.207
Ever smoking		1.380 (0.926, 2.058)	0.113
Alcohol consumption	Never drinking		
Current drinking		0.873 (0.597, 1.276)	0.484
Ever drinking		1.199 (0.764, 1.881)	0.431
Age groups	45–54		
55–64		1.618 (1.254, 2.089)	<0.001
≥65		2.375 (1.751, 3.221)	<0.001
FPG		1.009 (1.003, 1.014)	0.001
SBP		1.095 (1.002, 1.196)	0.044
TyG		1.040 (0.850, 1.273)	0.701

**Figure 2:**
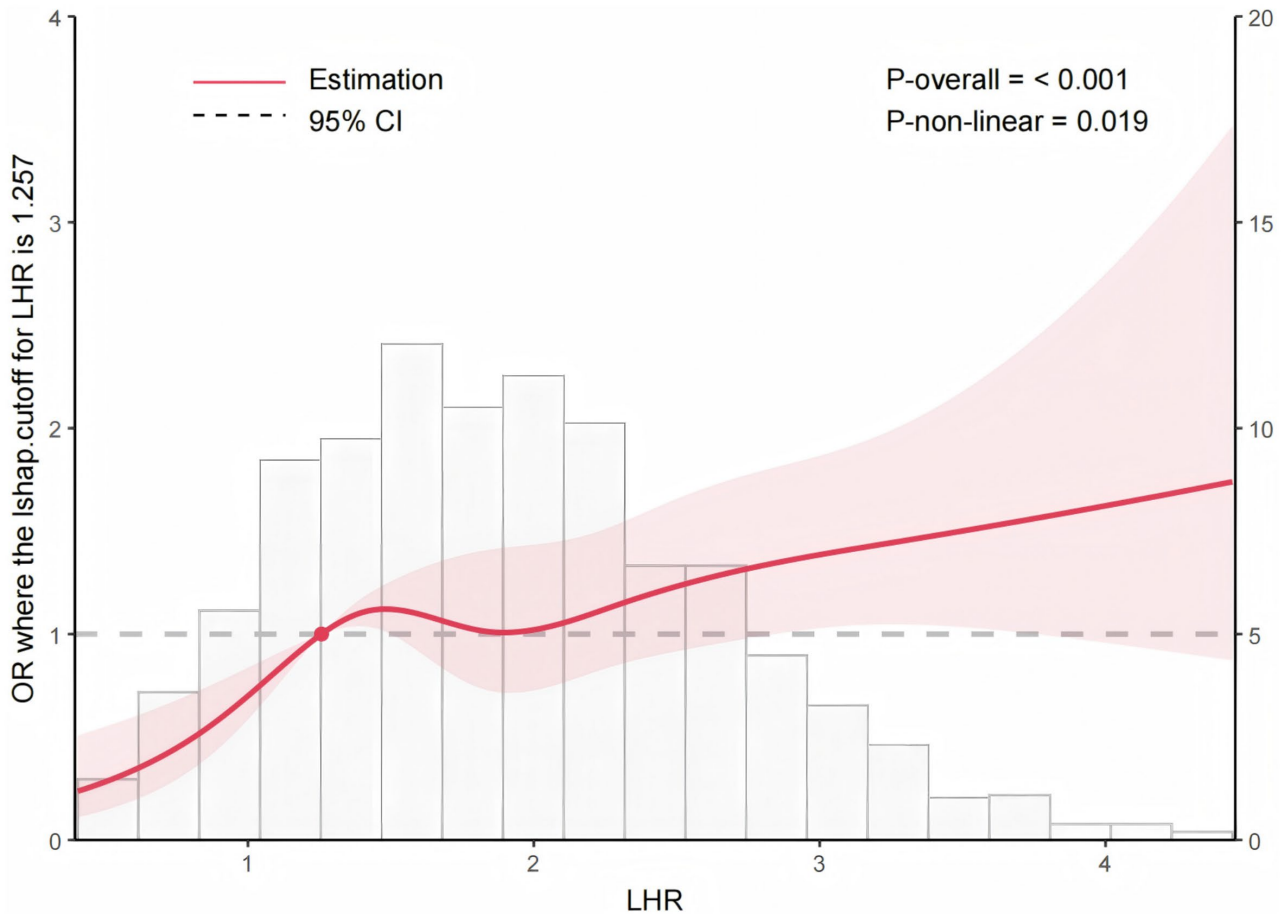
Restricted cubic spline analysis of the relationship between LHR and the risk of developing new carotid plaques. This figure demonstrated a significant nonlinear relationship between LHR and the risk of developing new carotid plaques (*p* = 0.019). The risk of new carotid plaques increased with higher LHR values, indicating a dose–response relationship.

Figure 2 illustrates the relationship between LHR and the risk of carotid artery plaque using RCS curves. The curve demonstrates a non-linear relationship between the two. The effect of low LHR values on carotid plaque risk is significant pro, but as LHR increases, the risk of developing carotid plaque rises sharply, with an optimal cut-off value of 1.257.

### 3.4 Logistic regression analysis of LHR groups on the development of carotid artery plaque

The logistic regression results show that compared with LHR ≤ 1.257, LHR > 1.257 increases 83.5% risk of new carotid plaques (OR = 1.835, 95% CI: 1.383–2.435, *p* < 0.001). In addition, compared with Q1 (LHR ≤ 1.36), Q2 (1.36 < LHR ≤ 1.84) increases 60.3% risk of new carotid plaques (OR = 1.603, 95% CI: 1.178–2.180, *p* = 0.003), and Q4 (LHR > 2.39) increases 103.0% risk of new carotid plaques (OR = 2.030, 95% CI: 1.484–2.778, *p* < 0.001) (Table 4).

**Table 4:** Logistic regression analysis evaluating the impact of LHR groups on the development of carotid artery plaque.

Characteristics	Unadjusted model		Adjusted model	
	Crude OR (95% CI)	*p* value	Adjusted OR (95% CI)	*p* value
Category				
LHR ≤ 1.257	Reference			
LHR > 1.257	1.800 (1.377, 2.354)	<0.001	1.835 (1.383,2.435)	<0.001
Quartering				
Q1 (LHR ≤ 1.36)	Reference			
Q2 (1.36 < LHR ≤ 1.84)	1.494 (1.113, 2.004)	0.007	1.603 (1.178, 2.180)	0.003
Q3 (1.84 < LHR ≤ 2.39)	1.280 (0.952, 1.721)	0.103	1.326 (0.969, 1.816)	0.078
Q4 (LHR > 2.39)	1.995 (1.491, 2.669)	<0.001	2.030 (1.484, 2.778)	<0.001

Adjusted model: adjusted for sex, smoking history, drinking history, age groups, SBP (per 1 mmHg for SBP), TyG and FPG.

### 3.5 Multivariate analysis of LHR and new carotid plaques in subgroups

Subgroup analyses revealed that the association between LHR and new carotid plaques varied across different demographic and clinical groups. In men, each unit increase in LHR was associated with a 58% higher risk of new carotid plaques (OR = 1.58, 95% CI: 1.24–2.02, *p* < 0.001). In women, the risk increase was 22% (OR = 1.22, 95% CI: 1.02–1.46, *p* = 0.027). Participants aged <60 years had a 41% higher risk (OR = 1.41, 95% CI: 1.16–1.73, *p* < 0.001), while those aged ≥60 years had a 28% higher risk (OR = 1.28, 95% CI: 1.05–1.56, *p* = 0.015). In the hypertension subgroup, each unit increase in LHR was associated with a 30% higher risk (OR = 1.30, 95% CI: 1.11–1.52, *p* = 0.001), and in the non-hypertension group, the risk increase was 44% (OR = 1.44, 95% CI: 1.07–1.93, *p* = 0.017). No significant association was found between LHR and new carotid plaques in the diabetes subgroup, while in the non-diabetes group, each unit increase in LHR was associated with a 39% higher risk (OR = 1.39, 95% CI: 1.19–1.63, *p* < 0.001) (Table 5).

**Table 5:** Multivariate analysis of the relationship between carotid artery plaque and LHR in subgroups^a^.

Subgroups	Adjusted OR (95%CI)	*p* value
Total	1.37 (1.19, 1.57)	<0.001
Men	1.58 (1.24, 2.02)	<0.001
Women	1.22 (1.02, 1.46)	0.027
<60 years	1.41 (1.16, 1.73)	<0.001
≥60 years	1.28 (1.05, 1.56)	0.015
Hypertension	1.30 (1.11, 1.52)	0.001
No hypertension	1.44 (1.07, 1.93)	0.017
Diabetes	/	
No diabetes	1.39 (1.19, 1.63)	<0.001

a The overall population was adjusted for sex, smoking history, drinking history, FPG, age groups, SBP, and LHR. Men group adjusted for age groups, FPG, BMI, LHR, SBP. Women group adjusted for age groups, FPG, SBP, and LHR. The <60-year-old group adjusted for sex, smoking history, drinking history, BMI groups, FPG, LHR, SBP. Those ≥60 years old were adjusted for sex, diabetes, hypertension and LHR. The hypertension subgroup was adjusted for sex, smoking, drinking, FPG, age groups, SBP, and LHR. The no hypertension group adjusted for sex, smoking history, age groups, FPG, SBP, LHR. The no diabetes group was adjusted for sex, smoking history, drinking, FPG, age groups, SBP, and LHR. SBP (per 1 mmHg for SBP).

## 4 Discussion

The primary objective of this study was to evaluate the association between the LHR and the incidence of new carotid plaques in a low-income rural population over a six-year period. This study found that the increase in LHR was associated with the risk of developing carotid plaques in a low-income rural population over a six-year period. The RCS curves suggested a non-line relationship between LHR and the risk of carotid plaques, and the optimal cut-off value was recommended to be 1.257. Our research shows robust results in the subgroup analyses. In summary, our study shows the association between LHR and the risk of carotid plaques, indicating that increased LHR may be expected to be a novel biomarker for carotid plaques. Our research provides new insights into the early identification of carotid plaques, suggesting that the LHR may be a potential biomarker for carotid plaques in Low-Income Rural Populations. Further research is needed to confirm our findings.

The relationship between the LHR and the development of carotid plaques has been explored in various studies and remained controversial. Previous research has consistently reported that LHR is a significant predictor of cardiovascular events and atherosclerosis (8, 9). Studies have found that the LHR predicted carotid intima-media thickness (CIMT) progression better than LDL-C or HDL-C alone, suggesting that LHR is a comprehensive marker of lipid-related cardiovascular risk (8). A high LHR was associated with an increased risk of sudden cardiac death, highlighting its potential as an emerging risk factor for severe cardiovascular outcomes (9). LHR was positively associated with CIMT and the presence of carotid plaques in male but not female patients with type 2 diabetes (10). LHR was a predictor of common carotid plaque in obese adults in Xinjiang, China, reinforcing its role in diverse population groups (11). Moreover, a higher LHR was associated with an accelerated development of carotid plaques in a routine physical examination cohort over a follow-up period of 1.05 years (13). However, the results of Hou et al. (19) and Nimkuntod and Tongdee (20) showed that the LHR had no significant predictive value for carotid plaque. Our findings are consistent with these studies in demonstrating the predictive value of LHR for carotid plaque formation. We found that each unit increase in LHR was associated with a 37% higher risk of developing new carotid plaques. This may be due to differences in the type of study and characteristics of the study population, and larger prospective cohort studies should be conducted in the future to validate the results of this experiment.

The association between the LHR and cardiovascular outcomes has been reported to exhibit gender-specific differences. Previous studies have explored the varying impact of lipid profiles on cardiovascular risk between men and women. The previous study found that the LHR was positively associated with CIMT and the presence of carotid plaques in male but not female patients with type 2 diabetes, indicating a stronger predictive value of LHR in men (10). The progression of carotid intima-media thickness was better predicted by LHR in men compared to women, suggesting a gender-specific difference in the predictive power of LHR for atherosclerosis (8). A study on obese adults in Xinjiang, China, found that the LHR was a predictor of carotid plaque in men, but the association was less pronounced in women (11). Moreover, in patients with coronary heart disease, a higher LHR was associated with a greater risk of carotid plaques in men compared to women (12). Our study corroborates these findings by demonstrating that the association between LHR and new carotid plaques is stronger in men than in women. Men showed a 58% higher risk of developing new carotid plaques for each unit increase in LHR, while women had a 22% higher risk. The mechanisms underlying these gender differences may involve hormonal influences and lifestyle factors. Estrogen in women promotes HDL formation and reduces LDL levels, providing a protective effect against atherosclerosis (21–23). This hormonal advantage might attenuate the impact of LHR on carotid plaque development in women. In contrast, men lack this hormonal protection and are more likely to engage in lifestyle behaviors such as smoking and alcohol consumption, which exacerbate the risk of atherosclerosis (14).

The relationship between age and the LHR in predicting cardiovascular outcomes has been documented in several studies. Previous research has consistently shown that aging is a significant risk factor for atherosclerosis and related conditions. The recent study demonstrated that a higher LHR was associated with an accelerated development of carotid plaques, with the effect being more pronounced in older adults (13). In a cohort of patients with coronary heart disease, older age groups exhibited a stronger association between high LHR and the risk of carotid plaques compared to younger age groups (12). The progression of carotid intima-media thickness was better predicted by LHR in older individuals, suggesting that age amplifies the predictive power of LHR for atherosclerosis (8). In a study on obese adults, observed that the association between LHR and carotid plaque was stronger in older participants compared to younger ones (11). Aligns with these findings, our study confirmed that the association between LHR and new carotid plaques is influenced by age. Participants aged under 60 years had a 41% higher risk of developing new carotid plaques for each unit increase in LHR, while those aged 60 years or older had a 28% higher risk. This indicates that while LHR is a significant predictor across all age groups, its impact is more substantial in younger individuals within our study population. The observed age-related differences may be attributed to various mechanisms. Younger individuals with high LHR might have more pronounced lipid metabolism disorders and early endothelial dysfunction, leading to a higher relative risk of developing carotid plaques compared to their older counterparts. In older individuals, the cumulative burden of various risk factors over time might overshadow the impact of LHR alone, resulting in a relatively lower incremental risk. Additionally, aging is associated with physiological changes such as arterial stiffness, reduced endothelial function, and increased oxidative stress, which contribute to atherosclerosis (24–26). These age-related changes may modify the impact of LHR on plaque development, suggesting that age-specific strategies might be necessary for effective cardiovascular risk management.

The relationship between the LHR, hypertension, and diabetes in predicting atherosclerosis has been a focal point of cardiovascular research. Previous studies have documented the interplay between these factors and the risk of developing carotid plaques. Kunutsor et al. highlighted that a higher LHR was significantly associated with an increased risk of cardiovascular events in individuals with hypertension (9). Li et al. (12) found that among patients with coronary artery disease, those with hypertension and a high LHR had a markedly higher risk of carotid plaques compared to non-hypertensive patients. In type 2 diabetic patients, a higher LHR was linked to greater carotid intima-media thickness and the presence of carotid plaques, particularly in men (10). A significant association between LHR and carotid plaque formation in the general Chinese adult population, with a stronger effect in hypertensive individuals (19). The previous study reported that in a routine physical examination cohort, higher LHR predicted carotid plaque development more effectively in individuals without diabetes compared to those with diabetes (13). Our study corroborates these findings, demonstrated a significant association between LHR and new carotid plaques in hypertensive individuals, reinforcing the role of hypertension in exacerbating the impact of LHR on atherosclerosis. However, our study did not find a significant association between LHR and new carotid plaques in the diabetes subgroup, which contrasts with some of the aforementioned studies. In the present study, we found that in the non-diabetes group, each unit increase in LHR was associated with a 39% higher risk of new carotid plaques. Further research is needed to elucidate the mechanisms by which diabetes interacts with lipid ratios to influence atherosclerosis, particularly in low-income rural populations.

Previous research has indicated that the relationship between lipid ratios and cardiovascular risk may not be strictly linear, suggesting more complex interactions at different levels of LHR. Kunutsor et al. identified a nonlinear association between LHR and the risk of sudden cardiac death, indicating that the risk increases more sharply at higher levels of LHR (9). A nonlinear relationship between LHR and the incidence of carotid plaques, with the risk rising significantly at higher LHR values in a cohort of routine physical examination participants (13). Another study found that the relationship between LHR and carotid plaque formation in patients with coronary heart disease exhibited nonlinear characteristics, with the most significant risk increase observed at higher LHR levels (12). Line with these findings, our study confirmed a significant nonlinear relationship between LHR and the risk of developing new carotid plaques. The restricted cubic spline analysis indicated that the risk of new carotid plaques increased nonlinearly with higher LHR values. This suggests that the impact of LHR on carotid plaque formation is not uniform across its range, with more substantial risk increases at higher levels of LHR. The exact mechanism that causes this nonlinear relationship is unknown and may be related to antioxidant and anti-inflammatory responses.

This study has several limitations that should be considered when interpreting the results. First, the study population was limited to a low-income rural area in Tianjin, which may affect the generalizability of our findings to other populations. This specific demographic might have unique characteristics and risk factors that differ from urban or more affluent populations, potentially limiting the applicability of our results to broader contexts. Second, the cohort was relatively homogeneous in terms of socio-economic status, which could influence the observed associations between LHR and carotid plaque development. The lack of diversity in the sample might mean that our findings are not fully representative of more varied populations with different lifestyle, genetic, and environmental influences. Future studies should aim to include a more diverse population sample, encompassing various socio-economic statuses, urban and rural settings, and different ethnic backgrounds. This would enhance the generalizability of the findings and provide a more comprehensive understanding of the relationship between LHR and carotid plaque development. Third, the six-year follow-up period, while providing valuable long-term data, may not capture the full dynamics of carotid plaque development. Intervals between follow-up assessments were long, potentially missing intermediate changes and the progression of atherosclerosis. Increasing the frequency of follow-up assessments would allow for a more detailed tracking of carotid plaque progression and the impact of changes in LHR over shorter intervals. This could help capture intermediate changes and provide a more nuanced understanding of the temporal dynamics involved. Fourth, there may be unmeasured confounding factors, such as dietary habits, physical activity levels, and genetic predispositions, that were not accounted for in the analysis. These factors could potentially influence the relationship between LHR and carotid plaque development. Comprehensive data collection, including a wider range of potential confounding factors, such as dietary intake, physical activity, and genetic information, would help to better isolate the specific effects of LHR on carotid plaque development and provide a more detailed context for interpreting the results. Finally, the study did not analyze the relationship between medication use and carotid plaque due to the small number of participants on medication (*n* = 13). Medication use could have a significant impact on lipid levels and cardiovascular outcomes, and its exclusion might have affected the results. Future studies should consider the impact of medication use on lipid levels and cardiovascular outcomes, ensuring that this variable is adequately measured and analyzed. Including medication use data would help to account for its potential confounding effects and provide a clearer picture of the relationship between LHR and carotid plaque development.

## 5 Conclusion

This study demonstrates a significant, non-linear relationship between the LDL-C/HDL-C ratio (LHR) and the long-term risk of developing carotid plaques in a low-income rural population in Tianjin, China. Over a six-year follow-up period, higher LHR levels were associated with an increased risk of carotid plaque formation, with a threshold of 1.257 identified as a critical cut-off point. The findings suggest that LHR could serve as a valuable and easily accessible biomarker for early detection and risk assessment of carotid plaques, especially in middle-aged and elderly populations. Given the high burden of atherosclerotic cardiovascular and cerebrovascular diseases globally, these results highlight the potential of using LHR as an efficient screening tool, particularly in resource-limited settings. The strong association between LHR and carotid plaque development across various subgroups further underscores its robustness as a predictor. This study provides essential evidence for public health initiatives focused on early intervention and prevention, advocating for the integration of LHR in routine clinical assessments to identify individuals at higher risk for cardiovascular diseases.

## Data Availability

The raw data supporting the conclusions of this article will be made available by the authors, without undue reservation.
