# Supplementary material for: Association between LDL-C/HDL-C ratio and long-term carotid plaque risk in middle-aged and elderly rural populations: a prospective population study
Source: Front Med (Lausanne). 2026 Feb 17;13:1771449. doi: 10.3389/fmed.2026.1771449 (PMC12953421; doi:10.3389/fmed.2026.1771449)
Supplement: Supplementary file 1 [file Table_1.DOCX]

Supplementary Material

Supplementary Table 1. Collinearity diagnosis

| Characteristics | Tolerance | Variance Inflation Factor |
| --- | --- | --- |
| TyG | 0.788 | 1.269 |
| FPG | 0.818 | 1.223 |
| LHR | 0.945 | 1.058 |
